# Adverse upgrading and/or upstaging in contemporary low-risk prostate cancer patients

**DOI:** 10.1007/s11255-022-03250-0

**Published:** 2022-07-15

**Authors:** Rocco S. Flammia, Benedikt Hoeh, Lukas Hohenhorst, Gabriele Sorce, Francesco Chierigo, Andrea Panunzio, Zhe Tian, Fred Saad, Costantino Leonardo, Alberto Briganti, Alessandro Antonelli, Carlo Terrone, Shahrokh F. Shariat, Umberto Anceschi, Markus Graefen, Felix K. H. Chun, Francesco Montorsi, Michele Gallucci, Pierre I. Karakiewicz

**Affiliations:** 1 Department of Maternal-Child and Urological Sciences, Sapienza Rome University, Policlinico Umberto I Hospital, Rome, Italy; 2 Cancer Prognostics and Health Outcomes Unit, Division of Urology, University of Montréal Health Center, Montréal, Québec Canada; 3 Department of Urology, University Hospital Frankfurt, Goethe University Frankfurt Am Main, Frankfurt am Main, Germany; 4 Martini-Klinik Prostate Cancer Center, University Hospital Hamburg-Eppendorf, Hamburg, Germany; 5 Division of Experimental Oncology/Unit of Urology, URI, Urological Research Institute, IRCCS San Raffaele Scientific Institute, Milan, Italy; 6 Department of Surgical and Diagnostic Integrated Sciences (DISC), University of Genova, Genova, Italy; 7 Department of Urology, University of Verona, Verona, Italy; 8 Department of Urology, Comprehensive Cancer Center, Medical University of Vienna, Vienna, Austria; 9 Department of Urology, University of Texas Southwestern, Dallas, TX USA; 10 Hourani Center for Applied Scientific Research, Al-Ahliyya Amman University, Amman, Jordan; 11 Department of Urology, Weill Cornell Medical College, New York, NY USA; 12 Department of Surgical Sciences, Sapienza Rome University, Rome, Italy

**Keywords:** Upgrading, Upstaging, Low risk, Radical prostatectomy, Prostate cancer

## Abstract

**Background:**

Upgrading and/or upstaging in low-risk prostate cancer (PCa) patients may represent an indication for active treatment instead of active surveillance (AS). We addressed contemporary upgrading and/or upstaging rates in a large population based-cohort of low-risk PCa patients.

**Materials and methods:**

Whitin the SEER database (2010–2015), NCCN low-risk PCa patients were identified across management modalities: radical prostatectomy (RP), radiotherapy (RT) and non-local treatment (NLT). In RP patients, upgrading and/or upstaging rates were assessed in logistic regression models.

**Results:**

Overall, of 27,901 low-risk PCa patients, 38% underwent RP vs 28% RT vs 34% NLT. RP patients were the youngest and harbored the highest percentage of positive cores and a higher rate of cT2a than NLT. At RP, 46.2% were upgraded to GGG ≥ 2, 6.0% to GGG ≥ 3 and 10.5% harbored nonorgan-confined stage (NOC, pT3-4 or pN1). Of NOC patients, 1.6% harbored GGG ≥ 3, 6.3% harbored GGG2 and 2.6% harbored GGG1. Of pT2 patients, 4.4% harbored GGG ≥ 3, 33.9% harbored GGG2 and 51.3% harbored GGG1. Age, PSA, percentage of positive cores and number of positive cores independently predicted the presence of NOC and/or GGG ≥ 3, but with low accuracy (63.9%).

**Conclusions:**

In low-risk PCa, critical changes between tumor grade and stage at biopsy vs RP may be expected in very few patients: NOC with GGG ≥ 3 in 1.6% and NOC with GGG2 in 6.3%. Other patients with upgrading and/or upstaging combinations will invariably harbor either pT2 or GGG1 that far less critically affect PCa prognosis.

## Introduction

North American and European guidelines recommend active surveillance (AS) in low-risk prostate cancer (PCa) patients, based on comparable long-term oncologic outcomes relative to active treatment ^1–4^. However, these guidelines are based on findings of historical studies ^5–8^ when conservative management rates (active surveillance/active monitoring/watchful waiting) were substantially lower than currently ^9,10^. Moreover, grading of PCa might have not been the same, since in contemporary patients the combination of systemic and target biopsy has shown to significantly reduce upgrading rates ^11–14^. Similarly, contemporary use of MRI prior to prostate biopsy decreased the rate of clinically insignificant PCa diagnoses at initial biopsy ^15–17^. In consequences historical data may not be applicable to contemporary patients. To address this void, we tested most contemporary upgrading and/or upstaging rates with specific focus on critical upgrading and/or upstaging, respectively, defined as GGG ≥ 3 and/or nonorgan-confined stage. We hypothesized that contemporary upgrading and/or upstaging rates were substantially lower than historical rate.

## Materials and methods

### Study population

The Surveillance, Epidemiology and End Results (SEER) database samples 26% of the USA and approximates the USA in terms of geographic and demographic composition, as well as cancer incidence^18^. Within the SEER database spanning years 2010–2015, we identified all non-metastatic patients, aged between 40 and 75 years, with histologically confirmed adenocarcinoma of the prostate, diagnosed at biopsy (International Classification of Disease for Oncology [ICD-O-3] code 8140 site code C61.9), who fulfilled the NCCN low-risk criteria (cT1c-T2a and biopsy Gleason grade group I and/or PSA < 10 ng/ml).

We excluded patients with number of biopsy cores < 10 or > 14, as well as cases with missing information (PSA, clinical T stage, biopsy Gleason grade group, number of positive prostate biopsy cores). This resulted in a cohort of 27,901 patients exposed to any treatment modalities: radical prostatectomy (RP), radiotherapy (RT) and non-local treatment (NLT). The main study group in whom upgrading and/or upstaging rates were examined consisted of 9355 RP patients. Here, we excluded those individuals with missing information on pathologic T stage and Gleason grade group (GGG). This resulted in 9126 assessable patients.

### Statistical analyses

In the first part of the analyses, patient characteristics were compared across treatment modalities (RP, RT, NLT). In the second part, we reported rates of upgrading and/or upstaging at RP. Upgrading was defined as change from GGG1 at biopsy to GGG ≥ 2 at RP. A stricter definition of upgrading relied on change from GGG1 at biopsy to GGG ≥ 3 at RP. Upstaging to nonorgan-confined stage (NOC) was defined as presence of extracapsular extension of the tumor (ECE, pT3a) or seminal vesicle invasion (SVI, pT3b) or pT4/pN1 stage at RP. Additionally, different combinations of upgrading and/or upstaging were computed and survival outcomes for these combinations were recorded. Moreover, we stratified upgrading and/or upstaging rates according to PSA (≤ 4.5 vs 4.5–5.8 vs > 5.8 ng/ml), percentage of positive cores (≤ 17 vs 17–33 vs > 33%), number of positive cores (≤ 2 vs 3–4 vs ≥ 5), age (< 60 vs > 60 years ) and race/ethnicity (Caucasians vs African-Americans vs Hispanic vs Asian vs others). Last, but not least, multivariable logistic regression models predicting two definitions of combined upgrading and/or upstaging were fitted and the accuracy of the models was calculated with previous methodology ^19,20^. All tests were two-sided with a level of significance set at *p* < 0.05 and R software environment for statistical computing and graphics (version 3.4.3) was used for all analyses.

## Results

### Baseline characteristics of the entire low-risk PCa patient cohort

Overall (Table 1), of 27,901 low-risk PCa patients 9,355 underwent RP (34%) vs 7820 underwent RT (28%) vs 10,726 underwent NLT (38%). RP patients were younger (59 vs 64 vs 63 years, *p* < 0.001), harbored higher median percentage of positive cores (25 vs 17 vs 14%, *p* < 0.001) and higher number of positive cores (3 vs 2 vs 2, *p* < 0.001) than RT or NLT counterparts. RP patients exhibited higher rates of cT2a than NLT (7.1 vs 5.4%), but not than RT patients (7.1 vs 7.1%). Finally, no differences in baseline PSA were recorded between RP vs RT vs NLT patients (5.1 vs 5.5 vs 5.4).

**Table 1:** Baseline characteristics of low-risk prostate cancer patients stratified according to management choice: radical prostatectomy (RP) vs radiation therapy (RT) vs non-local treatment (NLT)

	Overall, *N* = 27,901^1^	RP, *n* = 9,355 (34%)^1^	RT, *n* = 7,820 (28%)^1^	NLT, *n* = 10,726 (38%)^1^	*p* value^2^
Median age (years)	62 (57, 67)	59 (54, 64)	64 (59, 68)	63 (58, 68)	< 0.001
Age (category)					< 0.001
Younger (≤ 60 years)	11,636 (42%)	5,345 (57%)	2,511 (32%)	3,780 (35%)	
Older (> 60 years)	16,265 (58%)	4,010 (43%)	5,309 (68%)	6,946 (65%)	
Baseline PSA (ng/ml)	5.30 (4.40, 6.70)	5.10 (4.20, 6.40)	5.50 (4.50, 6.90)	5.40 (4.50, 6.80)	< 0.001
Clinical T stage					< 0.001
T1c	26,104 (94%)	8,693 (93%)	7,265 (93%)	10,146 (95%)	
T2a	1,797 (6.4%)	662 (7.1%)	555 (7.1%)	580 (5.4%)	
Biopsy cores (*n*)	12 (12, 12)	12 (12, 12)	12 (12, 12)	12 (12, 12)	< 0.001
Number of positive cores (*n*)	2 (1, 4)	3 (1, 5)	2 (1, 4)	2 (1, 3)	< 0.001
Percentage of positive cores (%)	17 (8, 33)	25 (10, 42)	17 (8, 33)	14 (8, 21)	< 0.001
Race–ethnicity					< 0.001
Caucasian	19,224 (68.9%)	6,722 (71.9%)	5,246 (67.1%)	7,256 (67.6%)	
African-American	4,210 (15.1%)	1,231 (13.2%)	1,426 (18.2%)	1,553 (14.5%)	
Hispanic	2,602 (9.3%)	904 (9.7%)	686 (8.8%)	1,012 (9.4%)	
Asian	1,193 (4.3%)	379 (4.1%)	285 (3.6%)	529 (4.9%)	
Unknown/other	672 (2.4%)	119 (1.3%)	177 (2.3%)	376 (3.5%)	

^1^Median (IQR) and n (%)

^2^Wilcoxon rank sum test and Pearson's Chi-squared test

### Upgrading and upstaging rates in low-risk prostate cancer patients

Overall (Table 2), 46.2% were upgraded to GGG ≥ 2 (4215/9126). Of those, 40.2% were GGG2 (3669/9126) vs 6.0% were GGG ≥ 3 (546/9126). Among those, 4.7% were GGG3 (433/9126) vs 1.3% were GGG4-5 (113/9126).

**Table 2:**
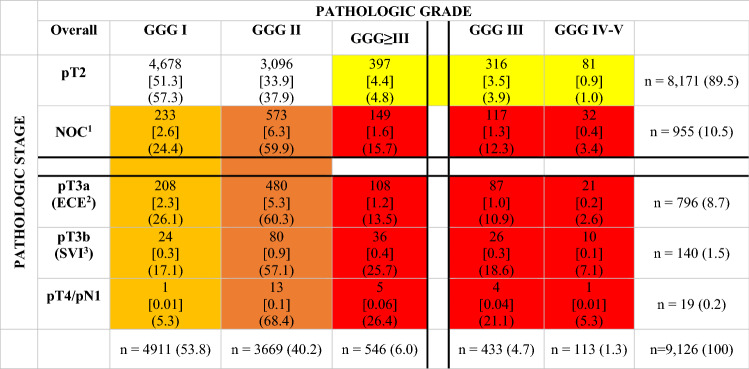
Cross-tabulation of final pathologic stage vs pathologic Gleason grade group (GGG) in low-risk prostate cancer (PCa) patients treated with radical prostatectomy (RP)

Color shade reflect different combinations of stage and grade at RP: white = pT2 and GGG1-2; yellow = pT2 and GGG≥3; lighter orange = NOC and GGG1; darker orange = NOC and GGG2; red = NOC and GGG≥3

^*^In each cell, the absolute number of individuals *x*, proportions out of total [*y*] and row proportions (*z*) are displayed

^1^Non-organ confined (NOC) was defined as the presence of pT3a or pT3b or pT4 or pN1

^2^Extracapsular invasion (ECE)

^3^Seminal vesicle invasion (SVI)

Overall (Table 2), 10.5% were upstaged to NOC (955/9126). Of those, 8.7% harbored ECE (796/9126) vs 1.5% harbored SVI (140/9126) vs 0.2% were upstaged to pT4 and/or pN1 (19/9126).

In GGG ≥ 3 patients (*n* = 149), 1.2% harbored ECE (108/9126), 0.4% harbored SVI (36/9126) and 0.06% were upstaged to T4 or pN1 (5/9126). In GGG2 patients (*n* = 573), 5.3% harbored ECE (480/9126), 0.9% harbored SVI (80/9126) and 0.1% were upstaged to T4 or pN1 (13/9126). In GGG1 patients (*n* = 233), 2.3% harbored ECE (208/9126), 0.3% harbored SVI (24/9126) and 0.01% were upstaged to T4 or pN1 (1/9126).

**Fig. 1:**
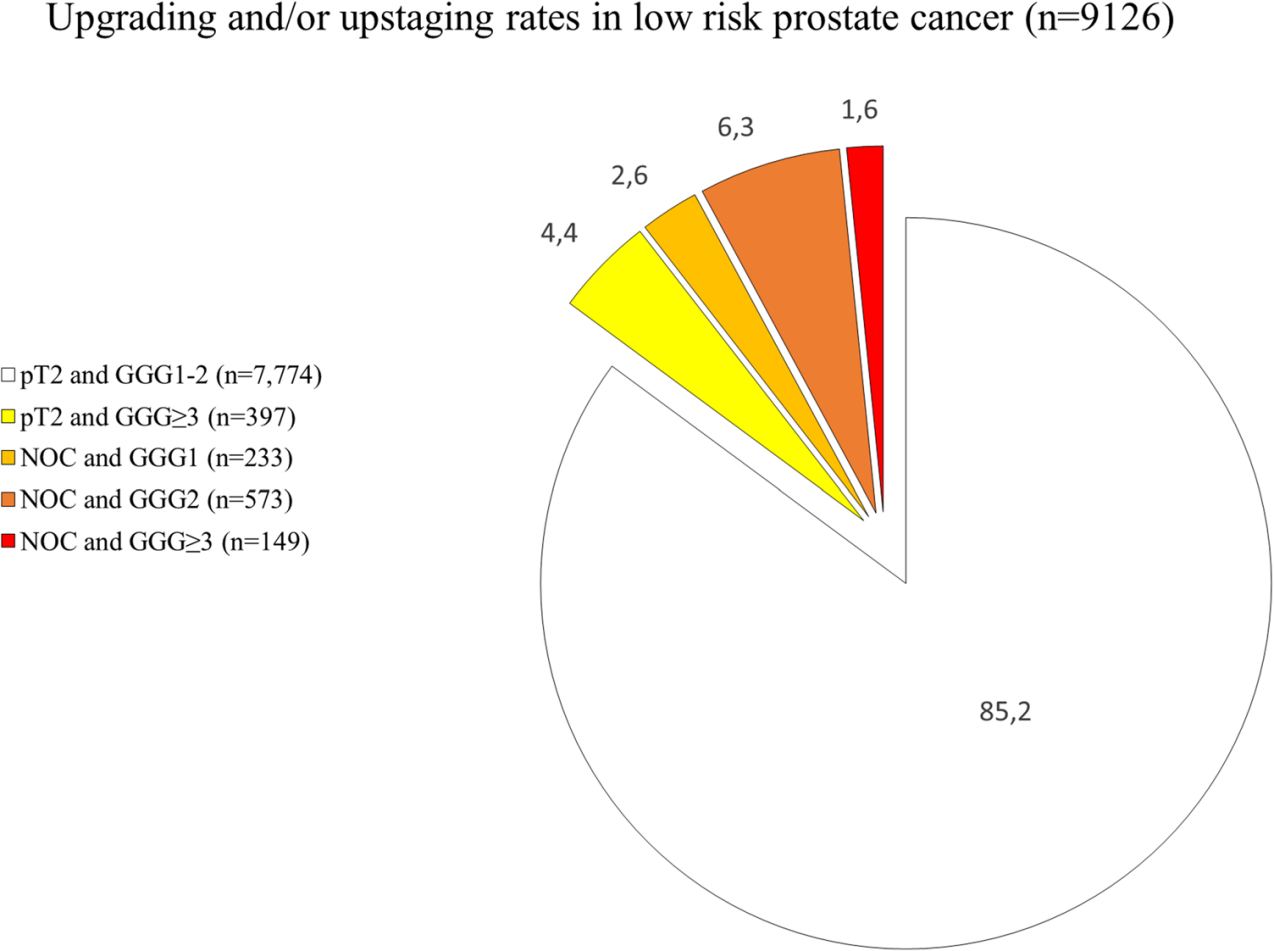
Pie chart displaying rates of different combinations of upgrading and/or upstaging in low-risk prostate cancer patients treated with radical prostatectomy (*n* = 9126). Pathologic stage was defined as pT2 vs nonorgan confined (NOC, pT3a or pT3b or pT4 or pN1). Pathologic tumor grade was define as Gleason grade group (GGG) 1–2 vs 2 vs ≥ 3

Among patients upstaged to NOC (*n* = 955), 1.6% harbored GGG ≥ 3 (149/9126), 6.3% harbored GGG2 (573/9126) vs 2.6% harbored GGG1 (233/9126). Conversely, of patients with organ-confined stage (pT2), 4.4% harbored GGG ≥ 3 (397/9126) vs 33.9% harbored GGG2 (3096/9126) and 51.3% harbored GGG1 (4678/9126). Different combinations of upgrading and/or upstaging were depicted in Fig. 1. Finally, detailed upgrading and/or upstaging rates according to patients characteristics are also tabulated (Supplementary Table 1).

### Model predicting upgrading and/or upstaging

For purpose of multivariable logistic regression modeling, we relied on two end points: (1) upgrading to GGG ≥ 2 and/or NOC stage at RP (48.7%; 4,448/9126); (2) upgrading to GGG ≥ 3 and/or NOC stage at RP (14.5%; 1,352/9126).

In model 1, age, PSA, clinical T stage, percentage of positive cores, number of positive cores, but not race/ethnicity were independent predictors of pT2 with GGG2 (Table 3). The accuracy of the model was 63.1% (95%CI 62.0–64.2%). In model 2, age, PSA, percentage of positive cores and number of positive cores, but not clinical T stage and race/ethnicity were independent predictors of NOC and/or GGG ≥ 3 (Table 3). The accuracy of the model was 63.9% (95%CI 62.3–65.6%).

**Table 3:** Multivariable logistic regression models predicting favorable upgrading/upstaging and unfavorable upgrading/upgrading in low-risk prostate cancer patients treated with RP

		Upstaging/upgrading (pT3–4 or pN1 or GGG ≥ 2), *n* = 4,448 (48.7%)^a^			Upstaging/upgrading (pT3–4 or pN1 or GGG ≥ 3), *n* = 1352 (14.5%)^a^		
Characteristic	*N*	OR^a^	95% CI^a^	*p* value	OR^a^	95% CI^a^	*p* value
Age (median, years)	9126	1.03	1.02–1.04	< 0.001	1.04	1.03–1.05	< 0.001
PSA (median, ng/ml)	9126	1.13	1.11–1.16	< 0.001	1.16	1.13–1.20	< 0.001
Clinical T stage *n* (%)							
T1c	8,478	–	–		–	–	
T2a	648	0.79	0.67–0.93	0.005	1.00	0.79–1.25	> 0.99
Number of positive cores *n* (%)							
≤ 2	4257	–	–		–	–	
3–4	2428	1.70	1.54–1.88	< 0.001	1.50	1.30–1.74	< 0.001
≥ 5	2441	2.34	2.11–2.59	< 0.001	2.15	1.87–2.46	< 0.001
Percentage of positive cores *n* (%)		1.02	1.02–1.02	< 0.001	1.02	1.01–1.02	< 0.001
Race/ethnicity *n* (%)							
Caucasian	6557	–	–		–	–	
African American	1197	1.09	0.97–1.24	0.16	1.01	0.84–1.19	0.94
Hispanic	885	0.95	0.83–1.10	0.51	0.99	0.81–1.21	0.93
Asian	370	1.16	0.94–1.43	0.16	1.18	0.89–1.55	0.24
Other	117	1.01	0.70–1.46	0.95	0.60	0.30–1.07	0.11
Accuracy (%)^b^		63.1	62.0–64.2		63.9	62.3–65.6	

^a^*OR* odds ratio, *CI* confidence interval

^b^Accuracy was computed as a C-index and bootstrapper 95%CI

### Survival differences in low-risk PCa patients according to pathologic stage and grade at RP

Overall, 12 deaths from PCa vs 139 deaths from other causes were recorded during 38,476 person-years of follow-up. After stratification according to different combinations of upgrading and/or upstaging definitions (NOC with GGG ≥ 3; NOC with GGG2; NOC with GGG1; pT2 with GGG ≥ 3; pT2 with GGG1–2), the following survival outcomes were recorded (Table 4).

**Table 4:**
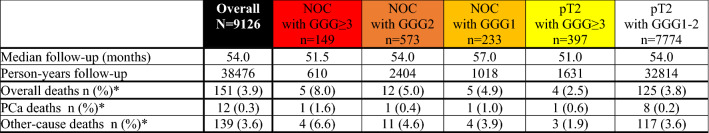
Survival outcomes were reported for low-risk prostate cancer (PCa) in the overall cohort of low-risk PCa patients treated with radical prostatectomy (RP), as well as according to stratification based on different combination of stage and grade at final pathological examination

Color shade reflect different combinations of stage and grade at RP: white = pT2 and GGG1-2; yellow = pT2 and GGG≥3; lighter orange = NOC and GGG1; darker orange = NOC and GGG2; red = NOC and GGG≥3

^a^*n* (%) = absolute number of deaths (person-time rate of deaths per 1000 person-year)

In NOC with GGG ≥ 3 (*n* = 149), one death from PCa vs four deaths from other causes were recorded during 610 person-years of follow-up. This resulted in 1.6% person-time PCa-specific death rate per 1000 person-years. In NOC with GGG2 (*n* = 573), 1 death from PCa vs 11 deaths from other causes were recorded during 2404 person-years of follow-up. This resulted in 0.4% person-time PCa-specific death rate per 1000 person-years. In NOC with GGG1 (*n* = 233), one death from PCa vs four4 deaths from other causes were recorded during 1,018 person-years of follow-up. This resulted in 1.0% person-time PCa-specific death rate per 1000 person-years. In pT2 with GGG ≥ 3 (*n* = 397), one death from PCa vs three3 deaths from other causes were recorded during 1631 person-years of follow-up. This resulted in 0.6% person-time rates of PCa-specific deaths per 1000 person-years. Finally, in pT2 with GGG1–2 (*n* = 7774), 8 deaths from PCa vs 117 deaths from other causes were recorded during 32,814 person-years of follow-up. This resulted in 0.2% person-time PCa-specific death rate per 1000 person-years.

## Discussion

We hypothesized that contemporary low-risk patients harbor lower rates of upgrading and/or upstaging than their historical counterparts due to important differences in diagnostics. We tested this hypothesis within a large contemporary cohort of low-risk PCa patients who underwent RP. Additionally we compared baseline characteristics of low-risk RP patients to their low-risk RT and NLT counterparts, to ensure absence of clinically meaningful differences.

First, regarding differences between low-risk RP vs RT vs NLT patients, RP patients harbored more aggressive PCa phenotype than their RT or NLT counterparts. Specifically, RP patients harbored a highest percentage of positive cores than RT and NLT patients (median, 25 vs 17 vs 14%, *p* < 0.001). Moreover, RP patients harbored a higher proportions of cT2a stage than NLT (7.1 vs 5.4%), but not RT (7.1 vs 7.1%) patients. In consequence, based on more unfavorable baseline PCa characteristics, it may be postulated that upgrading and/or upstaging rates recorded in RP patients, might be higher than those affecting RT or NLT patients. Unfortunately, this hypothesis cannot be formally tested since pathological staging only applied to RP patients and true PCa stage is unknown in those managed with RT and/or NLT.

Second, we identified low, albeit clinically meaningful rates of upgrading and/or upstaging. Upstaging from clinically favorable risk to pathologic NOC PCa represents the most important rate limiting factor, when conservative management strategy is implemented. Of 9126 low-risk PCa patients, 955 (10.5%) exhibited NOC stage. The largest subgroup consisted of pT3a (8.7%; 796/9126), substantially smaller subgroups harbored respectively pT3b (1.5%; 140/9126) and pT4 or pN1 (0.2%; 19/9126). Of those with NOC, 722 patients also harbored presence of Gleason pattern four or higher (GGG ≥ 2; 7.9%; 722/9126). Fortunately, most of those were GGG2 (6.3%; 573/9126) and of those, most had pT3a stage (5.3%; 480/9126) and only a minority harbored higher stage (1.0%; 93/9126). Conversely, only 149 of NOC patients harbored primary pattern 4 or higher (GGG ≥ 3, 1.6%, 149/9126) and within those a very marginal number were GGG4–5 (0.4%; 32/9126). Taken together, these observations indicate that contemporary low-risk PCa classification will critically misclassify 149 (1.6%; 149/9126) patients with NOC and primary Gleason pattern 4 or higher (GGG ≥ 3). Additional 573 (6.3%; 573/9126) individuals will be misclassified with less far-reaching implications, based on presence of exclusive secondary Gleason pattern 4 (GGG2) and NOC. This small patient group (1.6% plus 6.3%) represents the fatal flaw of low-risk group assignment based on PSA, clinical T stage and biopsy alone. This rate may possibly be reduced significantly with the use of MRI^21,22^.

Third, we tested whether upgrading and/or upstaging can be accurately predicted. Despite our best attempts and use of usual predictive modeling techniques the accuracy of predictions was low for both endpoints: 63.1% for NOC and/or GGG ≥ 2 and 63.9% for NOC and/or GGG ≥ 3. In consequence, observed rates of upgrading and/or upstaging cannot be accurately predicted using the available risk factors, at least in the current study. It may be argued that introduction of MRI findings may significantly increase the predictive ability of such a model. To the best of our knowledge, two nomograms relied on MRI findings in addition to clinical variables to predict NOC and/or GGG ≥ 3 in European^19^ and North American^25^ cohorts of low and intermediate risk PCa patients. All two nomograms exhibited an added benefit, when MRI findings were added to clinical variables. However, these results need to be replicated in external validation samples. Taken together, since the ability of MRI to improve prediction of upgrading and/or upstaging is still under debate^26^, other clinical tools, such as PSMA PET/CT scanning^27^, biomarkers^28^ (the prostate health index, urine PCA3, 4Kscore, TMPRSS2–ERG and ConfirmMDX) and IHC^29^ and genetic risk score^30^ may prove of value. However, large-scale contemporary testing of these tools have not been completed, especially in independent external validation samples that include sufficient numbers of low-risk patients^31^.

Fourth, we examined survival outcomes in the overall cohort of RP low-risk PCa patients with a median follow-up of 54 months. Expectedly, we recorded extremely low PCa mortality rates in these patients during 38,476 person-years of follow-up. Only 12 deaths were attributable to PCa. PCa-specific deaths ranged from 1 to 8, after stratification according to different combinations of upgrading and/or upstaging definitions. Based on person-time PCa-specific death rate per 1,000 person-years, only NOC with GGG ≥ 3 (1.6%) distinguished itself from values recorded for other combinations, including NOC with GGG2 (0.4%), NOC with GGG1 (1.0%), and pT2 with GGG ≥ 3 (0.6%). Nonetheless, all these combinations of upgrading and/or upstaging definitions invariably exhibited higher person-time PCa death rate for 1,000 person-years than pT2 GGG1–2 patients (0.2%) that represents the ideal candidate for AS. However, despite adequate median follow-up (54 month), the extremely low absolute number of PCa deaths undermined the ability to perform usual cancer-specific mortality rate comparisons. In consequence, consideration of person-years of follow-up and comparison of PCa person-time PCa death rates for 1,000 person-years is of essence. In agreement with our findings, Brooks et al. observed that adverse pathology (NOC and/or GGG ≥ 3) at the time of RP is highly associated with future development of distant metastasis and PCa-specific death among low and intermediate risk PCa patients from a single institutional prospectively maintained database (*N* = 428) with extensive follow-up (20 years)^32^.

The current study is not devoid limitations. The first and foremost limitation of the current study relates to the type of patients included in the analyses of upgrading and/or upstaging. Since biopsy required comparison to RP pathology, invariably such individuals represent RP patients. In consequence our findings are only directly applicable and mostly generalizable to similar patients. To address this limitation, we compared low-risk RP cohort to low-risk RT and NLT patients, with respect to age, race–ethnicity and clinical PCa characteristics. In those comparisons low-risk RP patients exhibited younger age and worse PCa characteristics. In consequence, RP low-risk PCa patients, at least in the current study, most likely reflect a less favorable phenotype of low-risk PCa. Second, an additional important limitation is lack of MRI data, since MRI represents a standard of care in the diagnostic workup of not only newly diagnosed PCa patients, but also of individuals at risk of PCa ^2,4^. Last but not least, SEER represents a retrospective data repository. Although, no prospective trial addressed the end points of the current study, several large institutional databases relied on prospective gathered data on biopsy and pathologic findings^33^. Such databases are undoubtedly better. However, they do not offer the same sample size of low-risk PCa patients as SEER.

## Conclusions

In low-risk PCa patients, critical changes between tumor grade and stage at biopsy vs RP may be expected in few patients: NOC with GGG ≥ 3 in 1.6% and NOC with GGG2 in 6.3%. Others patients with upgrading and/or upstaging combinations will invariably harbor either pT2 or GGG1 that far less critically affect PCa prognosis.

## Data Availability

All data generated for this analysis were from the SEER database (https://seer.cancer.gov/data/). The code for the analyses will be made available upon request.
